# Delay of total joint replacement is associated with a higher 90-day revision rate and increased postoperative complications

**DOI:** 10.1007/s00402-022-04670-4

**Published:** 2022-11-04

**Authors:** Dominik Emanuel Holzapfel, Matthias Meyer, Max Thieme, Stefano Pagano, Frederik von Kunow, Markus Weber

**Affiliations:** 1 Department of Orthopaedic Surgery, Medical Center, Regensburg University, Asklepios Klinikum Bad Abbach, Kaiser-Karl V.-Allee 3, 93077 Bad Abbach, Germany; 2 Department of Orthopaedic and Trauma Surgery, Barmherzige Brueder Regensburg Medical Center, Regensburg, Germany

**Keywords:** Surgical delay, Surgery postponement, Elective surgery, TJR, THA, TKA, Postoperative complications, Revision rate, COVID-19 pandemic

## Abstract

**Purpose:**

Delay of elective surgeries, such as total joint replacement (TJR), is a common procedure in the current pandemic. In trauma surgery, postponement is associated with increased complication rates. This study aimed to evaluate the impact of postponement on surgical revision rates and postoperative complications after elective TJR.

**Methods:**

In a retrospective analysis of 10,140 consecutive patients undergoing primary total hip replacement (THR) or total knee replacement (TKR) between 2011 and 2020, the effect of surgical delay on 90-day surgical revision rate, as well as internal and surgical complication rates, was investigated in a university high-volume arthroplasty center using the institute’s joint registry and data of the hospital administration. Moreover, multivariate logistic regression models were used to adjust for confounding variables.

**Results:**

Two thousand four hundred and eighty TJRs patients were identified with a mean delay of 13.5 ± 29.6 days. Postponed TJR revealed a higher 90-day revision rate (7.1–4.5%, *p* < 0.001), surgical complications (3.2–1.9%, *p* < 0.001), internal complications (1.8–1.2% *p* < 0.041) and transfusion rate (2.6–1.8%, *p* < 0.023) than on-time TJR. Logistic regression analysis confirmed delay of TJRs as independent risk factor for 90-day revision rate [OR 1.42; 95% CI (1.18–1.72); *p* < 0.001] and surgical complication rates [OR 1.51; 95% CI (1.14–2.00); *p* = 0.04].

**Conclusion:**

Alike trauma surgery, delay in elective primary TJR correlates with higher revision and complication rates. Therefore, scheduling should be performed under consideration of the current COVID-19 pandemic.

**Level of evidence:**

Level III—retrospective cohort study.

## Introduction

Considering current statistical findings, the population is aging as a result of demographic change [1]. In the year 2019, about 16.5% of the US population was older than 65 years [2]. Some projections assume an increase in this generation to a level of 23.4% in the year 2060 [3]. Also from a more moderate point of view, it can be concluded consequently that the number of degenerative diseases and the number of necessary TJRs in the population will increase. According to the 2021 annual report of the American Academy of Orthopedic Surgeons (AAOS) in the year 2020, about 254,295 TJRs were recorded in the American Joint Registry [4]. However, these data may be not complete. Unreported cases can be assumed here. Other evaluations like the US Healthcare Cost and Utilization Project (H-CUP) recorded a larger number of total joint arthroplasty procedures (599,500 THR and 715,200 TKR) for the year 2018 in the USA [5]. Some studies anticipate a drastic increase in hip and knee arthroplasty and in revision procedures by the year 2030 [6] with rising postoperative complications [7] and an estimated cost to Medicare of 50 billion US $ per year [8]. This means a big burden for the patients and a huge effort for the healthcare systems.

During the COVID-19 pandemic, delays of planned surgeries and especially of elective total joint replacement surgeries are becoming more and more frequent [9, 10]. This postponement of elective orthopedic surgeries additionally causes a major organizational and economical challenge [11, 12]. With the spread of the COVID-19 pandemic, there is a need to relearn how to budget and distribute resources and restructure patient care [13]. Setting priorities in favor of emergency and trauma surgery a massive decline in elective primary TJR as well as in revision TJR services was observed [9, 14]. A survey from the European Hip Society (EHS) and the European Knee Associates (EKA) showed a drastic reduction in arthroplasty surgeries of 82.6% and TJR delay rates of 50.7% during the COVID-19 pandemic [15]. Studies report on the dropping of 30,000 primary TJR and 3000 revision arthroplasty procedures in the USA per week [16]. The consequences of these developments have been unknown so far.

In the literature delays, acute trauma surgery correlated with increased postoperative mortality and showed a trend toward higher postoperative complications [17, 18]. For elective surgeries, prolonged waiting time has demonstrated an adverse effect on the functional outcome of THR procedures [19]. To our knowledge, no correlation between delay after primary elective TJR and increased complications or increased revision rates has been described so far.

The objective of the presented paper was to assess the effect of postponing elective primary TJR on revision and complication rates in a university high-volume arthroplasty center. We first quantified the amount of delay after elective TJR. As the primary question of this study we asked, if there was a positive correlation of postponement of elective TJR procedures and a higher revision rate (90-day and 60-day revision rate). In addition, the correlation of delay in surgery and postoperative transfusion rate and surgical and internal complications was investigated using the institute’s joint registry and data of the hospital’s administration. Finally, we investigated possible confounders correlating with a higher revision rate after elective primary TJR.

## Materials and methods

**Fig. 1:**
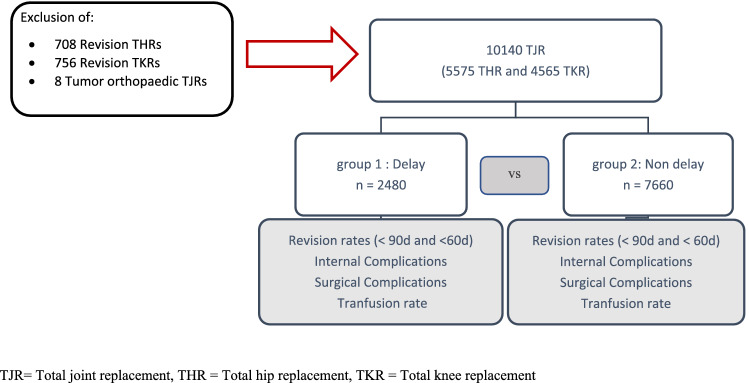
Flowchart of the study population

In a retrospective search, data of 10,140 consecutive patients undergoing elective primary total hip or knee replacement in a German Orthopedic University Hospital were identified. Patient data from the time period from January 2011 to December 2020 was retrieved from the in-house database (ORBIS; Agfa healthcare). Only elective primary THR or TKR surgeries were included in the study. Acute trauma cases, such as proximal femoral neck fractures, but also revision surgery or periprosthetic joint infections were excluded. From this population, 2480 patients with preoperative postponement of at least one day were selected, using the OPS-codes (Operation and Procedure Code) and individual case numbers. In contrast, 7660 patients could be identified who did not show preoperative delay, and the operation was performed as initially scheduled. If the surgery was performed at least one day later than initially scheduled, then this was defined as delay. Further patient data such as age, sex, BMI, ASA-Score (American Society of Anesthesiologists), operating time, hospital frailty risk score (HFRS) [20], internal and surgical complications, transfusion rates (yes/no), and reoperation rate < 90 days and < 60 days were evaluated using the institute’s joint registry and the data of the hospital’s administration system from 2011 until 2020 (Fig. 1). Complications were categorized in internal complications (cardiac complications: myocardial infarction and heart rhythm disorders; pulmonal complications: pneumonia and pulmonary edemas; renal complications: renal insufficiency and electrolyte derangement) and surgical complications (fractures, wound healing disturbance, and mechanical complications). The survey of the HFRS was conducted with the retrospective assignment of individual ICD-10 codes to each patient. These were matched with the 109 ICD-10 codes, which are characteristic for frailty and which are allocated to the respective severity of frailty in the form of point scores as defined by Gilbert et al. [20]. In summation, the maximal achievable score is 173.2 points [20].

All operations were performed in a single high-volume arthroplasty university center. All patients received the same standardized treatment protocol for THR or TKR, respectively. Cementless THR was performed in the lateral decubitus position. A minimally invasive anterolateral approach was used [21]. Press-fit acetabular components and cement-free stems of one single manufacturer (Pinnacle cup, Corail stem or Trilock stem; DePuy Synthes, J & J Medical Devices, Warsaw, IN) were used in all THR procedures. Cemented TKR was performed through a medial parapatellar approach. Cemented components of one single manufacturer (PFC Sigma; DePuy Synthes, J & J Medical Devices) were used in all TKRs. No patella resurfacing was performed.

For statistical analysis, continuous data are presented as mean (standard deviation). Group comparisons were performed by two-sided *t* tests. Absolute and relative frequencies were given for categorical data and compared between groups by Chi-square tests. The endpoints of the study were tested on 5% significance level. Multivariable logistic regression analyses were conducted to assess whether preoperative delay in primary elective total hip and knee arthroplasty independently correlates with postoperative revision rate while controlling for other variables such as operative time, type of surgery, sex, age, ASA classification, or frailty. IBM SPSS Statistics 26 (SPSS Inc, Chicago, IL, USA) was used for analysis.

## Results

**Fig. 2:**
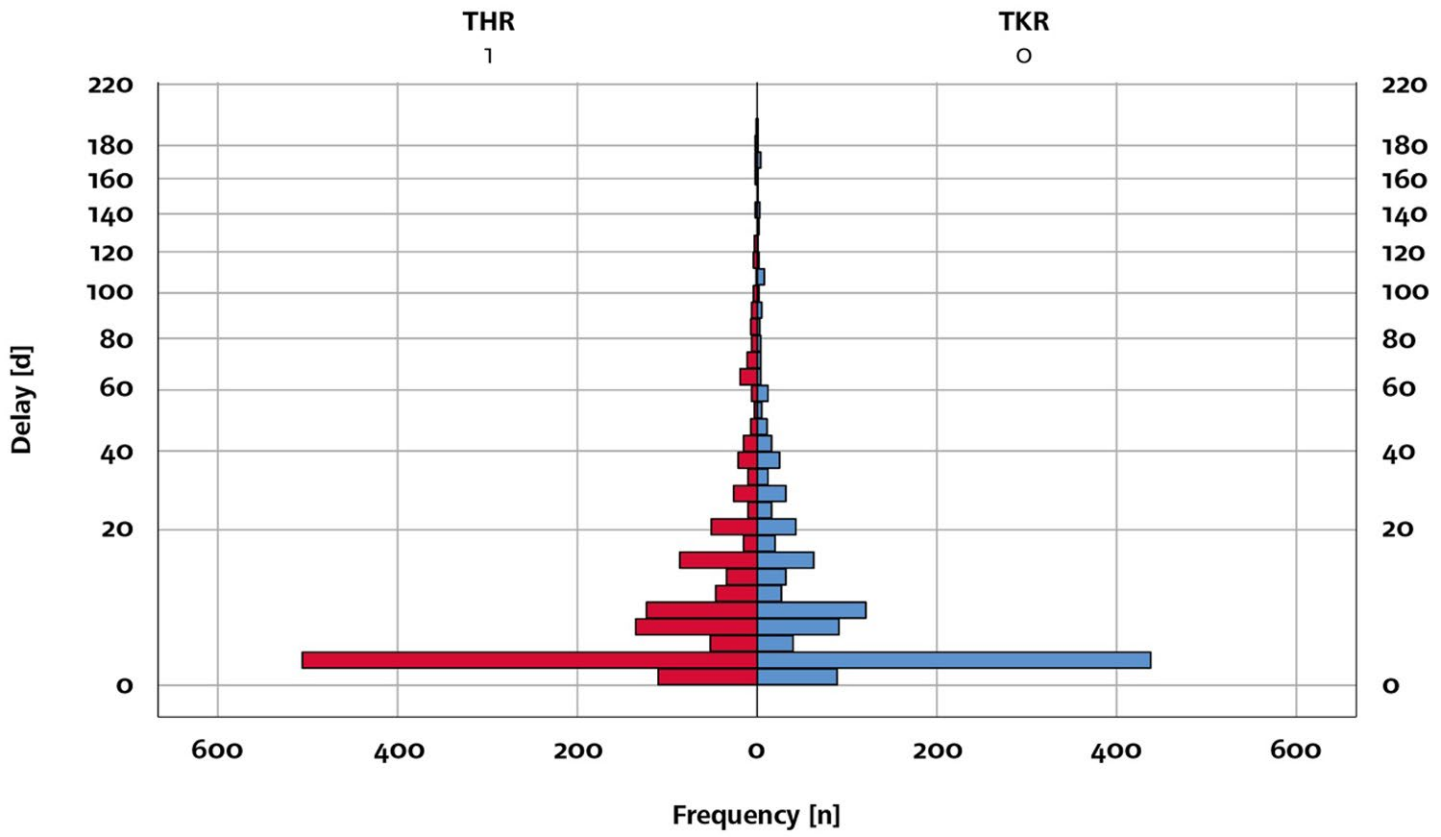
Frequency of delay of elective TJR. The frequency (= *n*) of the delay of elective TJR (d = days)

In the analysis of our in-house database (ORBIS; Agfa healthcare), we found data of 10,104 patients with condition after successful total joint arthroplasty in a time period from January 2011 to December 2020. Of these population, 55% (*n* = 5.575) people had undergone a hip replacement surgery and 45% (*n* = 4.565) a knee replacement surgery. The demographic characteristics of the study population are listed in Table 1. Among this population, there were 2480 patients with preoperative postponed elective surgeries, of which could be subdivided into 1335 delayed THR and 1145 delayed TKR surgeries. The mean of the delayed days until surgery for all TJRs was 13.5 days (SD: 29.6 days, THRs: 13.1 days, SD: 29.0; TKRs: 13.9 days, SD: 30.3, Fig. 2).

**Table 1:** Demographics of the study group

	No delay *n* = 7660	Delay *n* = 2480	Total *n* = 10,140	*p* value
Sex				
Female	57.2%	58.9%	57,6%	0.12
Male	42.8%	41.1%	42,4%	
THA	55.4%	53.8%	55%	0.19
TKA	44.6%	46.2%	45%	
ASA				
I	13.0%	12.0%	12.7%	0.02
II	57.1%	54.9%	56.5%	
III	29.7%	32.6%	30.5%	
IV	0.2%	0.4%	0.3%	
Age (years)	66.1 (10.7)	66.0 (10.8)	66.1 (10.7)	0.54
BMI (kg/m^2^)	29.0 (5.5)	29.1 (5.3)	29.1 (5.4)	0.70
HFRS	1.1 (2.0)	1.1 (1.9)	1.1 (2.0)	0.89
Operative time (min)	76.2 (30.9)	77.6 (28.7)	76.5 (30.4)	0.05
Length of stay (days)	8.9 (3.0)	10.0 (5.0)	9.1 (3.6)	< 0.001

Age, BMI, HFRS, operative time and length of stay are specified in mean (standard deviation)

*THA* total hip arthroplasty, *TKA* total knee arthroplasty, *ASA* American Society of Anesthesiologists, *BMI* body mass index, *HFRS* Hospital frailty risk score

**Fig. 3:**
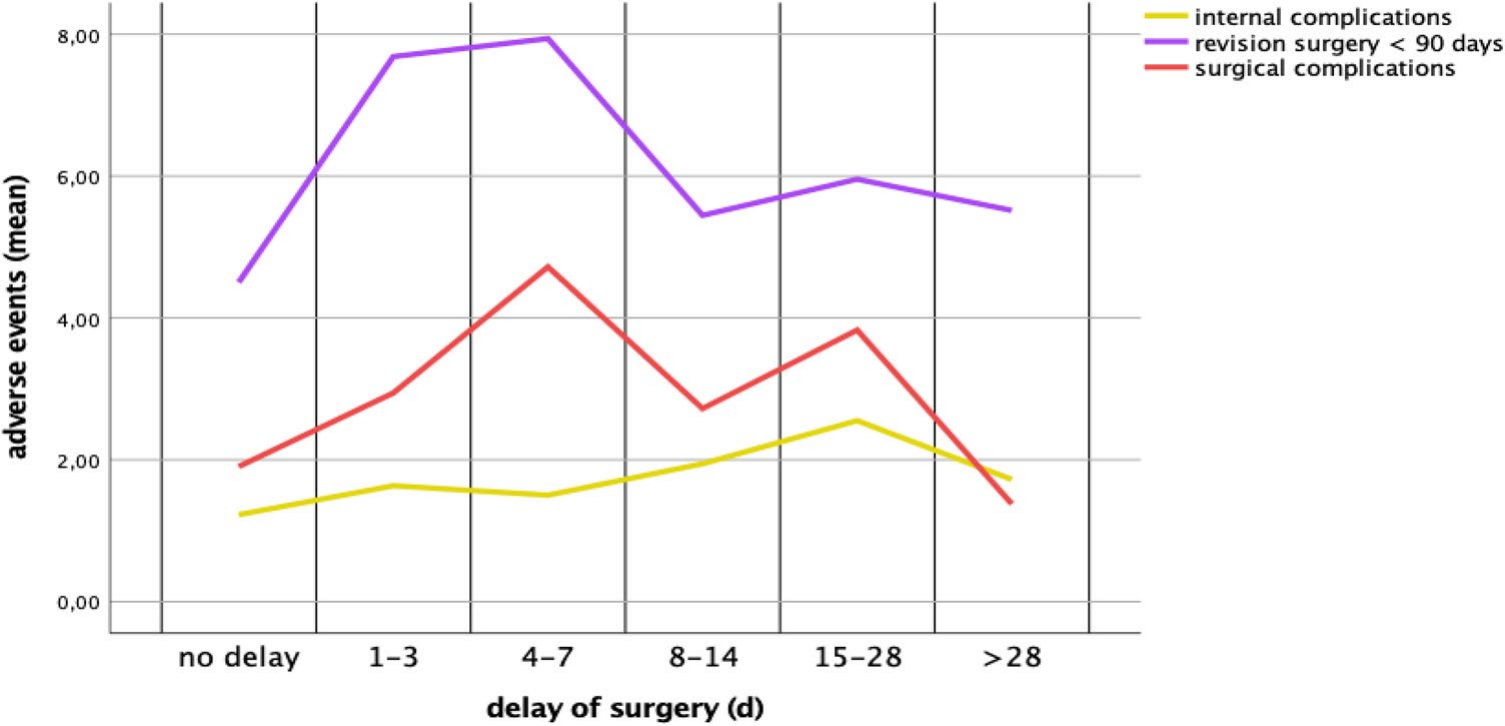
Adverse events in correlation to preoperative delay of elective TJR. Means of adverse events in correlation to delay of elective TJR in days

In the univariate analysis, delay in elective primary TJR correlated with a higher revision rate < 90 days with 7.1% (175/2480) than on-time surgery with 4.5% (345/7660; *p* < 0.001). Similarly, the rate of revisions < 60 days was higher after delay in elective primary TJR with 6.6% (164/2480) compared to on-time surgery with 3.9% (300/7660; *p* < 0.001). Internal complications after delay in elective primary TJR were with 1.8% (44/2480) higher than on-time TJRs with 1.2% (94/7660) (*p* value < 0.001). Surgical complications after delay in elective primary TJR showed even higher percentages of 3.2% (79/2480) in contrast to the on-time TJRs of 1.9% (146/7660) (*p* value < 0.001). Postoperative transfusion was higher after postponed TJRs with 2.6% (64/2480) in comparison with TJRs without delay with 1.8% (141/7660) (*p* value < 0.001). In addition to this results, individual univariate analyses of delay in elective primary THR and TKR were also broken down (Table 2). Considering the time of delay in days, a longer delay did not correlate with higher complication rates (internal/surgical complications, revisions < 90 days, Fig. 3).

**Table 2:** Results of univariate analysis with Pearson’s Chi-quadrat test of delayed TJR

TJRs	No delay *n* = 7660	Delay *n* = 2480	Population *n* = 10,140	*p* value
Univariate analysis with Pearson’s Chi-Quadrat-Test of TJR				

THRs	No delay *n* = 4240	Delay *n* = 1335	Total *n* = 5575	*p* value
Total hip replacements				
Revisions < 90 days	4.5% (191)	**7.0%** (93)	5.1% (284)	** < 0.001**
Revisions < 60 days	4.1% (173)	**6.4%** (86)	4.6% (259)	** < 0.001**
Internal complications	1.1% (47)	**2.3%** (31)	1.4% (78)	** < 0.001**
Surgical complications	2.2% (92)	**3.9%** (52)	2.6% (144)	** < 0.001**
Transfusion rate (yes/no)	2.0% (83)	2.7% (36)	2.1% (119)	0.103

TKRs	No delay *n* = 3420	Delay *n* = 1145	Total *n* = 4565	*p* value
Total knee replacements				
Revisions < 90 days	4.5% (154)	**7.2%** (82)	5.2% (236)	** < 0.001**
Revisions < 60 days	3.7% (127)	**6.8%** (78)	4.5% (205)	** < 0.001**
Internal complications	1.4% (47)	1,1% (13)	1.3% (60)	0.539
Surgical complications	1.6% (54)	2.4% (27)	1.8% (81)	0.840
Transfusion rate (yes/no)	1.7% (58)	2.4% (28)	1.9% (86)	0.106

The bold values highlight the significant data

Internal complications: cardiac complications like myocardial infarction and heart rhythm disorders, pulmonal complications like pneumonia and pulmonary edemas, renal complications like renal insufficiency or electrolyte derangement. Surgical complications: fractures, wound healing disturbance and mechanical complications. Transfusion rate (yes/no). The data of the sections no delay, delay and total are presented in percentage (%) and number (*n*)

*TJR* total joint replacement, *THR* total hip replacement, *TKR* total knee replacement

Even in multivariable analyses, delay in elective primary TJR showed a significant higher correlation with revision surgeries < 90 days [OR 1.42; CI (1.18–1.72); *p* < 0.001] and < 60 days [OR 1.59; CI (1.30–1.94); *p* < 0.001] than non-delayed surgeries. Other parameters such as ASA [OR 1.58; CI (1.34–1.85): *p* < 0.001], operative time [OR 1.01; CI (1.01–1.02): *p* < 0.001], and HFRS [OR 2.19; CI (1.61–2.97): *p* < 0.001] revealed an additional effect on the probability of occurrence of required revision surgery < 90 days. The results held true for 60-day revision rates (Table 3).

**Table 3:** Multivariate analysis showing the effect of preoperative delay of elective TJR on operative revision rate < 90 and < 60 days

	OR (CI 95%)	*p* value
Multivariate analysis with logistic regression		
Revisions < 90 days		
Delay	**1.42** (1.18 – 1.72)	** < 0.001**
ASA	**1.58** (1.34 – 1.85)	** < 0.001**
Age	1.01 (0.99 – 1.02)	0.191
Sex	0.89 (0.73 – 1.08)	0.220
Operative time	**1.01** (1.01 – 1.02)	** < 0.001**
TKR	0.92 (0.76 – 1.11)	0.390
HFRS	**2.19** (1.61 – 2.97)	** < 0.001**
Revision < 60 days		
Delay	**1.59** (1.30 – 1.94)	** < 0.001**
ASA	**1.58** (1.33 – 1.87)	** < 0.001**
Age	1.01 (0.99 – 1.02)	0.071
Sex	0.87 (0.71 – 1.07)	0.185
Operative time	**1.01** (1.01 – 1.02)	** < 0.001**
TKR	0.88 (0.72 – 1.08)	0.210
HFRS	**2.35** (1.72 – 3.22)	** < 0.001**

Multivariate calculations via logistic regression analyses to be aware of confounding variables

The bold values highlight the significant data

*OR* odds ratio, *CI* confidence interval, *ASA* American Society of Anesthesiologists, *TKR* total knee replacement, *HFRS* Hospital frailty risk score

In summary, the present study found a correlation between delay in elective primary TJR and increased 90-day revision rates, internal and surgical complications, and transfusion rates.

## Discussion

In the present study, we evaluated the effect of postponed elective surgery on revision and complication rates in elective TJR in a high-volume arthroplasty center. Analyzing a consecutive cohort of over 10,000 patients, delay in elective primary TJR correlated with increased revision rates within the first 90 days and similarly with higher internal and surgical complications and transfusion rates.

As described in the literature, it is recommended that surgical treatment of femoral neck fractures should be performed as early as possible within a range of 6–24 h in acute trauma care [22–24]. Looking at the postoperative mortality, morbidity, and postoperative complication rates, there is a significant correlation with a surgical delay of more than 24 h [17, 18, 22–26, 31–34]. There are even reports that the relative risk of death is up to 4.5-fold higher, if surgery does not occur until 24 h after admission [35]. Several studies confirm especially the rise of internal and surgical complication rates with a surgical delay of traumatic proximal femoral fractures of more than 24 h [26, 29, 36, 37]. One study even showed an increase in the relative risk of lethality and early revision by about one third in case of delay of more than 24 h [29]. Another study showed that patients with proximal femur fracture and a longer preoperative waiting time for surgery had an increased rate of early needed surgical revision with 6.4% [29]. It can be seen that our elective outcomes regarding revision rates are comparable to these values.

In the field of elective surgery, to our knowledge, there are only few studies showing an association of surgery postponement and complication rates. A study group in the sector of elective spine surgery found that patients with concomitant diseases and surgical delays showed an increased coherence with postoperative complications, like deep venous thrombosis and pulmonary embolism, return to the operating room, sepsis, cerebrovascular accident, progressive renal insufficiency, prolonged ventilator time, urinary tract infection, pneumonia, and wound infections. The deep wound infections in this paper were for example significantly higher for postponed surgeries with 3.2% compared with 1.0% for on-time surgeries. [38]. In elective spine surgery, a correlation between delays and negative patient outcomes could also be observed [39]. Looking at the above-mentioned rates of deep wound infections in delayed elective spine surgery, the rates are also comparable to our results concerning the revision rates of delayed surgeries, although there were slightly higher rates in our data. This shows that postponement of elective surgeries has negative effects.

As the number of elective total joint arthroplasty has risen even before the COVID-19 pandemic [6], a pronounced waiting time for elective joint replacement surgery could be observed due to an increasing demand [9]. With the developments and necessary measures in the context of the pandemic, the waiting time for elective total joint replacement surgery is reaching a peak. Even patients with severe symptoms have to be postponed due to limited resources for elective surgery. A study by Pietrzak et al. confirmed that patients whose elective TJA surgery was postponed during the COVID-19 pandemic experienced more pain and worse function [40]. Additionally, considering the costs for the healthcare system in the literature demonstrated that a postponement of elective operations causes a high financial burden [41, 42]. Furthermore, there is research showing that end-stage osteoarthritis causes a higher postoperative need for opioid analgesia and results in a worse outcome in terms of revisions and readmission rates after TJA [43]. In particular, even psychosocial consequences are to be expected in this context [44].

Given the current evolution of the COVID-19 pandemic, the primary need is to properly allocate resources (staff, beds, ICU beds, economic resources, post-discharge care, etc.) [45] and contain the pandemic spread. However, given the continuing and recurring constraints, secondarily in the long term, it is also necessary to find strategies and ways to allow elective surgery to avoid the far-reaching consequences that arise from surgical delays. Plans and models must be drawn up to deal with these consequences from a medical and financial point of view. Meanwhile, there are already research foci dealing with the perioperative management of elective surgery in times of the COVID-19 pandemic [46, 47]. Perhaps, this study can help to initiate more research in this area of elective surgery to minimize the risk of surgical revisions and postoperative complications.

Looking at the covariates in the multivariable correlation calculations, we could see that ASA, HFRS, and operative time also had an effect on revision rates < 90 days and < 60 days. While operative time and ASA showed a significant effect on surgical complications, ASA, age, and HFRS had a significant effect on internal medicine complications. In addition, there was a statistically significant effect of ASA, age, operative time, and HFRS on postoperative transfusion rates. These results are not surprising and confirm data from the known literature.

The present study certainly has some limitations. One of them is the model of a database study and the related retrospective design, which might favor a potential bias. We tried to reduce this analyzing a consecutive series of patients over a period of almost 10 years. Second, the current available data are limited to the data entered in our joint registry and coding program. Consequently, the resulting quality of information may be inherently limited. Other interesting parameters were not assessed and therefore not available. In particular, it would be interesting to know which complications occur more frequently and have the highest relevance when surgery is postponed. In this regard, our data were also limited, so we had to pool the individual complications to prove an effect. Another interesting point would be the detection of the different preoperative reasons for the postponement of surgeries. Unfortunately, this information was not collected in our database, so that it could not be utilized. However, it might have an impact on the results whether the scheduled surgery was delayed due to administrative or medical reasons. This subject could be content of further research projects.

The strengths of the present study are the big study cohort with over 10,000 patients and the monocentric design including a highly standardized surgical procedure with a standardized postoperative physiotherapeutic rehabilitation program avoiding potential confounders.

## Conclusion

The initial study objective could be successfully confirmed by demonstrating that there is a significant association of preoperative delay of elective TJR with revision surgery rates < 90 and < 60 days and also with internal and surgical complications and transfusion rates. This context is an important factor in long-term assessment and organization of elective surgeries with respect to changing demographics and the current COVID-19 pandemic.

## Data Availability

The data that support the findings of this study are available from the corresponding author upon reasonable request.
